# Process Control of Multistep Surface Functionalization on Hydroxyethyl Starch Nanocapsules Determines the Reproducibility of the Biological Efficacy

**DOI:** 10.1021/acs.biomac.4c00490

**Published:** 2024-10-22

**Authors:** Marie-Luise Frey, Svenja Morsbach, Matthias Domogalla, Volker Mailänder, Kerstin Steinbrink, Katharina Landfester

**Affiliations:** † Max Planck Institute for Polymer Research, Ackermannweg 10, Mainz 55128, Germany; ‡ Dermatology Department, University Medicine of the Johannes Gutenberg-University Mainz, Langenbeckstr. 1, Mainz 55131, Germany; § Department of Dermatology, University Hospital Münster, University of Münster, Von Esmarch-Strasse 58, Münster 14948, Germany

## Abstract

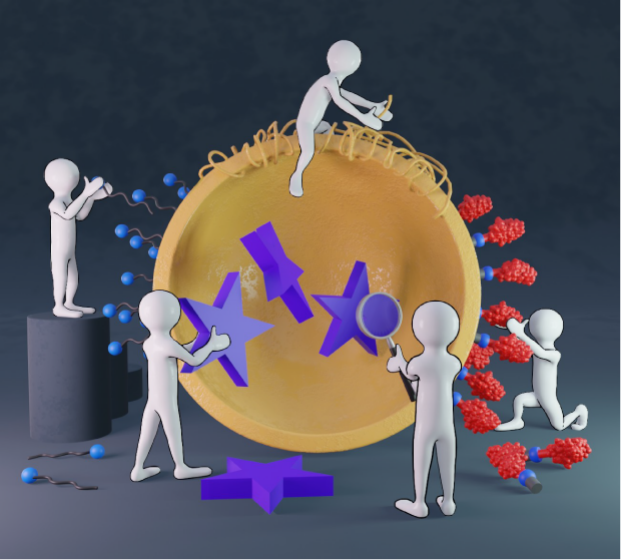

Nanocarrier synthesis is highly process-dependent, leading to potential batch-to-batch variability if it is not controlled at each step. This variability affects the reproducibility of subsequent biomodification, resulting in unpredictable biological effects, particularly for bioactive molecules such as interleukin-2 (IL-2). Inconsistent conjugation can lead to variable treatment outcomes and severe side effects. Therefore, precise control of each synthesis step is critical for ensuring a consistent quality and biological performance. Our study demonstrates that dividing nanocarrier synthesis into smaller, controlled steps improves reproducibility. Using this method, we achieved highly reproducible, concentration-dependent growth of CTLL-2 cells with hydroxyethyl starch (HES) nanocapsules functionalized with defined amounts of IL-2. We believe that such detailed, stepwise control in nanocarrier synthesis enhances batch consistency, improving the clinical applicability of the drug delivery systems.

## Introduction

As early as the 1980s, the cytokine IL-2 as a T cell and natural killer cell activator found its first use and is a well-characterized molecule.^1,2^ The administration of high-dose interleukin-2 (IL-2) was approved by the FDA as a medication for patients suffering from solid tumors such as renal cell carcinomas and melanomas (Proleukin). In addition, low-dose IL-2 has been shown to activate regulatory T cells and is therefore now being investigated as an immunotherapeutic agent to tackle autoimmune pathologies.^3^ Thus, IL-2 functionalization of nanocarriers will allow targeting of specific T cell subpopulations depending on the amount of coupled IL-2. However, in the case of combinations of IL-2 with nanosystems, the biological effect is dependent on the characteristics of the nanomaterial, the covalent or noncovalent addition to the nanocarrier, and a variety of process factors and therefore has to be well-monitored. One example is represented by the T cell targeting with IL-2-functionalized nanocapsules.^4^

Reproducibility of the desired biological effect is decisive for the therapeutic success of a drug, and therefore, a constant quality of the product is essential.^5^

The constant quality of any kind of molecular system is achieved by meticulously complying with every individual step of the processes of synthesis, purification, sterilization, and storage/distribution.^6^ In standard operating procedures (SOPs), synthesis processes are described in great detail, and guidelines for implementation and exact documentation are established, which leads to a high degree of consistency of product batches.^7^ However, the application of standardized protocols for the production of nanocarriers for drug delivery is not common.^8−10^ An even greater issue is the use of characterization protocols, which makes it challenging to determine if one is dealing with a reasonably comparable type of nanocarrier system. This seems surprising, considering that nanocarrier syntheses and characterization require many steps, each of which is highly process-dependent, demanding detailed documentation.^11^

From a chemical point of view, a defined biomodification of the surface of a nanocarrier is very challenging, as it depends on many factors that have been determined in earlier synthesis steps, such as size, surface charge, functional groups, or properties of the material itself.^12−14^ Hence, from a biological point of view, maintaining a constant biological response of biomodified nanocarriers must consequently depend on the previously performed synthesis steps.

To understand the parameters of the synthesis that are essential for a defined biological effect, in our model system, we modified the surface of hydroxyethyl starch (HES) nanocapsules with IL-2 and split the synthetic process into many small steps. We first synthesized the nanocapsules in an inverse miniemulsion and then transferred them into an aqueous medium. Afterward, we surface-functionalized the nanocapsules with dibenzocyclooctyne (DBCO) groups, enabling a copper-free alkyne–azide click reaction with azide-modified IL-2. We analyzed the size of the nanocapsules under different conditions and tested the use of different surfactants for water transfer. Next to the analysis of the size, FTIR spectroscopy demonstrated the chemical stability of nanocapsules. The detection of the DBCO groups by a fluorescent assay allowed adjustability of these surface groups.^15^ The functionalization of IL-2 and the binding to the nanocapsule were verified, and the biological activity was confirmed.

The defined binding of IL-2 onto nanocapsules is exceedingly desirable, since the biological effect of IL-2, which is the growth and differentiation of T cells, depends critically on the concentration present.^16,17^ Variations of IL-2 concentrations may therefore have severe effects on the intended immunological response.^2,4^

## Experimental Part

### Materials

All chemicals and reagents were purchased from commercial suppliers and used without further purification. Ampuwa water (sterile and pyrogen-free water, Product No. 1088813) and hydroxyethyl starch (HES, *M*_w_: 200 kDa, substitution degree 0.5, 3%), dissolved in an isotonic sodium chloride solution, were obtained by Fresenius Kabi (Germany). Sulforhodamine (SR101, Product No. S7635), 2,4-toluene diisocyanate (TDI, 95% Product No. T39853), dimethyl sulfoxide (DMSO, anhydrous, > 99%, Product No. 276855), sodium dodecyl sulfate (SDS, LiChropur, ≥ 99.0% (Art.No. 71726), Stains-all (Art.No: E9379), and Dulbecco’s phosphate-buffered saline (DPBS, without CaCl_2_ and MgCl_2_, Art.No. D8537) were obtained by Sigma-Aldrich (USA). Cyclohexane (HPLC grade) was purchased from VWR Chemicals. Cy5 Oligo was obtained by IBA Lifesciences (Germany) dissolved in aqueous solution at a concentration of 0.1 nm/μL, and Lutensol AT50 was obtained by BASF (Germany). DBCO-PEG_4_-NHS ester (Art.No. CLK-A134-100, purity >95%) was purchased from Jena Bioscience GmbH (Germany). NHS-PEG_4_-azide was obtained from Thermo Fisher Scientific Inc. (USA). Aqua ad iniectabilia (Art.No.: 03710653) was obtained from B. Braun (Germany). IL-2 (Art.No. CRI100XX) was purchased from Cell Sciences Inc. (USA). Sodium hydroxide solution, Volumetric, 0.1 M NaOH (0.1 N), Honeywell Fluka, was purchased by Fisher Scientific (USA). FITC-labeled anti-IL-2 antibody (Art.No.: 500305) was purchased from Biolegend (USA). Human IL-2 ELISA Kit was purchased from BD Biosciences (USA) (Product No. 550611). Anthracene azide and the block copolymer poly((ethylene-*co*-butylene)-*b*-(ethylene oxide) P(E/B)-*b*-EO) used as surfactant were synthesized according to previously published procedures.^18,19^

### Syntheses and Purification

#### Synthesis of HES Nanocapsules

The nanocapsules were prepared adopting previously published procedures from Baier et al. using an inverse miniemulsion process.^20^ Cyclohexane was dried over a molecular sieve (5 Å) before use for at least 1 day.

1400 mg of an aqueous HES solution (200 kDa, 0.5 substitution degree, 3%) was mixed with 1 mg of sulforhodamine 101 (SR101) or 200 μL Cy5 Oligo (0.1 nmol/μL) and 20 mg of sodium chloride (dispersed phase). The solution was covered with aluminum foil and was stirred for 5 min at room temperature until all components were completely dissolved. 100 mg of the surfactant P(E/B-*b*-EO) was dissolved in 7.5 g of dry cyclohexane under shaking and added at once to the dispersed phase. The mixture was pre-emulsified by vigorous stirring for 1 h at room temperature. Afterward, the emulsion was subjected to ultrasound for 3 min (70%, 20 s pulse, 10 s pause on a Branson Sonifier W-450-Digital with a 1/2′ tip) and subsequently placed in an oil bath at 25 °C equipped with a stirrer. Under stirring at 700 rpm, a mixture of 30 mg P(E/B-*b*-EO) and 100 mg TDI in 5.0 g dry cyclohexane was added dropwise with a syringe. The emulsion was stirred at 25 °C for 24 h.

#### Redispersion of HES Nanocapsules into Aqueous SDS Solution

5 mL of the nanocapsule dispersion was distributed over three 2 mL Eppendorf tubes, 1.667 mL each, and centrifuged for 30 min at 4000 rpm. The supernatant was replaced with the same amount of fresh dry cyclohexane, and the procedure was repeated. The pellets were finally redispersed in 500 μL of fresh cyclohexane and added dropwise into 5 mL of aqueous SDS solution (0.1 wt %) over a time period of 5 min under constant sonication in an ultrasonic bath (Bandelin Sonorex RK52H from Bandelin electronic Berlin, 160 W, 0.6 L). The aqueous dispersions were placed on a stirring plate, and the cyclohexane was evaporated at room temperature under vigorous stirring at 1000 rpm.

#### Functionalization of HES Nanocapsules with DBCO-PEG_4_-NHS Ester and Quantification of the DBCO Groups on the Nanocapsule Surface

The aqueous nanocapsule dispersion (solid content of ca. 0.1 wt %) was centrifuged at 4000 rpm for 30 min, and the supernatant was replaced once with the same amount of Ampuwa water (sterile and pyrogen-free water). The dispersion was again centrifuged at 4000 rpm for 30 min, and the supernatant was removed. The pellet was then resuspended in one-tenth of the original volume. This procedure led to an increase of the solid content from 0.1 to 1.0 wt %. 10 mg of DBCO-PEG_4_-NHS ester was dissolved in 500 μL dry DMSO. DMSO was dried over a molecular sieve (4 Å) before use. In order to compare the degree of functionalization, different amounts of DBCO were added to the dispersion. The amounts added varied between 3.3 mg of DBCO per mL of dispersion (165 μL of DBCO stock solution in DMSO (10 mg/500 μL)) and 0.04 mg/mL of dispersion. The mixture was stirred in a 4 mL glass vial overnight at room temperature. Afterward, the dispersion was centrifuged at 8000 rpm for 30 min, and the supernatant was replaced with the same amount of fresh Ampuwa water. The procedure was repeated with 5000 rpm centrifugation speed.

After the functionalization and purification of nanocapsules, the attached DBCO groups were quantified by an anthracene azide assay. Therefore, anthracene azide was freshly dissolved in DMSO (1.7 mg/mL), and corresponding molar amounts were added to of the DBCO-functionalized HES nanocapsules. The following amounts of anthracene azide were added to 25 μL of functionalized nanocapsules: A) (3.3 mg/mL DBCO): 17.1 μL anthracene azide, B) (1.1 mg/mL DBCO) 5.68 μL anthracene azide, C) (0.4 mg/mL DBCO): 2.84 μL anthracene azide, D) (0.2 mg/mL DBCO): 1.42 μL anthracene azide, and E) (0.04 mg/mL DBCO): 2.84 μL anthracene azide (1:10 dilution of stock solution). Furthermore, blank samples were prepared by mixing the corresponding amounts of anthracene azide (A–E) with 25 μL of water each and without nanocapsules. The total volume was adjusted with DMSO to 50 μL for each sample. The same procedure was followed with supernatants. The calibration was performed, mixing 16.5 μL of DBCO-PEG_4_-NHS ester (10 mg/500 μL DMSO) with 68.1 μL anthracene azide (1.70 mg/mL DMSO) (5.079 × 10^–7^ mol) and adding 15.4 μL DMSO to obtain a total volume of 100 μL. This first concentration was diluted 1:1 with DMSO, to obtain a calibration in concentrations from 3.3 mg/mL to 1.61 × 10^–3^ mg/mL. All samples were subjected to reaction for 1 h on a shaker at room temperature in the dark. After incubation, the samples were vortexed and a 1:10 dilution (90 μL DMSO + 10 μL sample) and a 1:100 dilution (99 μL DMSO + 1 μL sample) were pipetted in duplicates into the wells of a 96-well plate (Greiner Bio-one GmbH, Germany), and the fluorescence was measured on the Infinite M1000 plate reader from Tecan, Austria (Ex.: 370 nm, Em.: 414 nm). For data calculation, the average concentration of all replicates and dilutions was determined.

#### Functionalization of IL-2 with NHS-PEG_4_-Azide

Phosphate buffer was prepared by dissolving 1.78 g of Na_2_HPO_4_ × 2 H_2_O and 1.38 g of NaH_2_PO_4_ × H_2_O in 100 mL of Ampuwa water each. For pH 7.6, 13.0 mL of NaH_2_PO_4_ × H_2_O solution was mixed with 87.0 mL of the Na_2_HPO_4_ × 2 H_2_O solution. By addition of 90 mL sterile water and addition of 5 mL of each of the phosphate solutions, a phosphate buffer with a concentration of 50 mM and a pH of 7.6 was obtained.

1 mg portion (6.67 × 10^–8^ mol) of IL-2 was dissolved in 1 mL of freshly prepared phosphate buffer (50 mM, pH 7.6). The buffer was directly added to the IL-2 vial, which was vortexed and transferred to an Eppendorf tube (1.5 mL). 107 μL NaOH (0.1 M) was added to this mixture to readjust the pH of the mixture. 100 mg of NHS-PEG_4_-azide was dissolved in 1 mL of dry DMSO, and a 3-fold molar excess (2 × 10^–7^ mol, 7.77 μL (1:10 dilution)) was added to 1 mL of IL-2 solution. The mixture was stirred at room temperature for 2 h. To prepare the IL-2-functionalized nanocapsules as described here, usually, 2 mg of IL-2 needs to be functionalized. Therefore, the same procedure can be repeated for another 1 mg of IL-2 in a separate Eppendorf tube. In the end, both IL-2 mixtures could be combined for purification. For purification, the mixture was dialyzed against phosphate buffer for 2 days at 4 °C to remove residual azide linker. After purification, the functionalized IL-2 was concentrated with a centrifugal filter (Amicon centrifugal filters 3 kDa, Merck Millipore) to 2 mL, and the final concentration was determined by a Pierce 660 nm Assay (Thermo Fisher Scientific) according to manufacturer’s instructions.

#### Clicking of Azide-Functionalized IL-2 to DBCO-Modified HES Nanocapsules

In order to compare the relationship between the biological function and IL-2 concentration, different amounts of IL-2 were added to the DBCO-functionalized nanocapsules. IL-2 was added according to the calculated number of DBCO groups per nanocapsule in three different ratios (1:1, 1:10, and 1:100). Therefore, to fully functionalized DBCO-HES nanocapsules (functionalized with 3.3 mg/mL DBCO-PEG_4_-NHS ester), 1538 μL IL-2 (1:1), 153.8 μL IL-2 (1:10), and 15.38 μL IL-2 (1:100) were added to 350 μL nanocapsule dispersion each. The dispersions were then stirred at 4 °C for 3 days. Afterward, they were dialyzed against Ampuwa water (Visking Cellulose dialysis tube, Carl Roth, MWCO 14 kDa) and centrifuged twice at 6000 rpm for 30 min to remove residual IL-2, to concentrate the dispersion, and to maintain the sterility of water.

#### Purifications and Concentration by Centrifugation

Purification of nanocapsules with or without centrifuge filters was performed on a Sigma 3K30 centrifuge by Sigma, Germany, in a speed range from 4000 to 6000 rpm (1446 g–3254 g).

### Methods and Procedures

#### Light Scattering

Dynamic light scattering (DLS) measurements at a fixed angle of 90° were performed in triplicate on Malvern Zetasizer Nano S90 (Malvern Panalytical GmbH, Kassel, Germany).

For concentration determination, average sizes and size distributions were measured by multiangle dynamic light scattering at 20 °C using an instrument from ALV GmbH consisting of a goniometer and an ALV-5000 multiple-tau full digital correlator with 320 channels. As a light source, a helium–neon laser (JDS Uniphase with a single mode intensity of 25 mW operating at a laser wavelength of 632.8 nm) was used. Measurements were performed in quartz light scattering cuvettes (inner diameter 18 mm, Hellma, Müllheim), and dispersions were filtered dust-free with a 0.45 μm LCR filter prior to the measurements.

#### Infrared Spectra (FTIR)

Infrared spectral measurements were performed on a SpectrumBX FTIR spectrometer from PerkinElmer equipped with a diamond ATR unit.

#### SDS Quantification on Nanocapsule Samples

This procedure was adopted from previously published procedures.^21^ Briefly, 1 mg of stains-all dye was dissolved in 1 mL of isopropanol/water (1:1). 1 mL portion of the dye solution was mixed with 1 mL of formamide and 18 mL of water. Then, 200 μL of the mixed dye solution was mixed with 1–2 μL of a nanocapsule sample or SDS standard. The absorbance was measured at 438 nm in 96-well plates.

#### Labeling of IL-2-Modified HES Nanocapsules with the FITC-Anti-IL-2 Antibody

20 μL of IL-2-modified HES nanocapsules (1 wt %) was diluted with 180 μL of PBS buffer, and 20 μL of FITC-anti-IL-2 antibody (0.5 mg/mL) was added. The sample was vortexed and incubated for 30 min in the dark. Afterward, the dispersion was centrifuged 4 times at 20000 g for 15 min each, and the supernatant was replaced with the same amount of fresh PBS. In the end, the dispersion was concentrated to 30 μL by removal of the supernatant in the last centrifugation step and resuspension of the pellet in 30 μL of PBS buffer. The modified nanocapsules were subjected to FCCS measurements.

#### Fluorescence Correlation Spectroscopy (FCS) and Fluorescence Cross-Correlation Spectroscopy (FCCS)

FCS and FCCS measurements were performed using a commercial setup LSM 880 microscope (Carl Zeiss, Jena, Germany). For excitation of FITC and Cy5 Oligo, an argon ion laser (488 nm) and a He/Ne-laser (633 nm) were used. The excitation light was focused into the sample by a C-Apochromat 40×/1.2 W (Carl, Zeiss, Jena, Germany) water immersion objective. The fluorescence light was collected with the same objective and, after passing through a confocal pinhole, directed to a spectral detection unit (Quasar, Carl Zeiss). The detected emission range was in the spectral range of 500–553 nm for FITC and in the range of 642–696 nm for Cy5 Oligo. For calibration of the detection volumes, Alexa Fluor488 and Atto Fluor643 were used.

The measurements were performed in an eight-well polystyrene-chambered coverglass (Laboratory-Tek, Nalge Nunc Internation, Penfield, NY, USA). For all solutions, a series of 20 measurements with a total duration of 3 min were performed. Beside the measurement of the HES nanocapsules, HES capsules were incubated with Cy5 Oligo to exclude an adsorption of the dye on the surface of nanocapsules. For FCCS measurements, IL-2-modified HES nanocapsules were incubated with FITC-labeled anti-IL-2 antibodies.

#### Time-of-Flight Secondary Ion Mass Spectrometry (ToF-SIMS)

ToF SIMS measurements were performed on IONTOF TOF.SIMS5 NCS (IONTOF GmbH, Münster, Germany) with Bi3@30 keV primary ion pulses, 0.11 pA, on a field of view of 200 × 200 μm^2^. Cycle time: 150 μs (mass range: 1–2070 u).

#### Fluorescence Intensity Measurements

Fluorescence intensity measurements were operated in 96-well plates on an Infinite M1000 plate reader from Tecan, Austria.

#### Matrix-Assisted Laser Desorption and Ionization Time-of-Flight (MALDI-TOF)

MALDI-TOF measurements were performed on a Bruker Reflex II MALDI-TOF mass spectrometer (Bremen, Germany) equipped with a N_2_-laser (λ = 337 nm). α-Cyano-4-hydroxycinnamic acid was used as a matrix dissolved in water/acetonitrile with trifluoroacetic acid as a cationizing agent.

#### Sandwich ELISA

ELISA was conducted according to protocols of BD Biosciences OptEIA (USA) and after previously published procedures.^22^ The IL-2 capture antibody was incubated on 96-well microplates at 4 °C overnight. After a washing step with washing buffer (1× PBS supplemented with 0.05% Tween-20) to remove a residual capture antibody, unspecific epitopes were saturated by incubation with blocking buffer (1× PBS supplemented with 10% fetal calf serum (FCS)) for 1 h. Afterward, the nanocapsules with different IL-2 modifications were incubated on the prepared microplates at dilutions ranging from 1:1000 to 1:10,000,000 for 2 h at room temperature. After washing the microplates again with washing buffer, an enzyme-linked detection antibody (1:250) and the streptavidin horseradish peroxidase reagent (Streptavidin-HRP conjugate 1:250) were added, and the mixture was incubated for another 1 h at room temperature. Then, plates were washed again with washing buffer to remove residual reagents, and the substrate 3,3′,5,5′-tetramethylbenzidin was added. After 20–30 min, enzymatic activity was stopped by addition of stop solution (1 M H_3_PO_4_ and 1 N H_2_SO_4_), and extinction was detected with a photometer (Medel 450, Bio-Rad Laboratories, München, Germany Software: KC Junior Bio-Tek Instruments GmbH, Bad Friedrichshall, Germany) at 450 nm.

#### CTLL-2 Proliferation Assay

CTLL-2 proliferation assessed by means of ^3^H-thymidine assay upon HES-IL-2 NCs addition was performed and evaluated by previously published procedures.^4,22^ Briefly, CTLL-2 cells were cultured together with HES-IL-2 NCs at different concentrations.

As a reference, Proleukin was added to another group of CTLL-2 cells. The plates were pulsed with ^3^H-thymidine, and proliferation was analyzed by radioactivity measurements.

## Results and Discussion

For the encapsulation of multiple hydrophilic components into a defined polymeric nanocapsule with high loading capacity, high encapsulation efficiency, and tunable functionality, the approach via the inverse miniemulsion process is one of the most suitable strategies. However, the synthesis and the subsequent functionalization procedure required multiple steps and can be split into three main steps, starting (1) with the formation of the inverse miniemulsion and the polymerization at the interface to obtain the nanocapsules, followed by (2) the transfer of the nanocapsules from the organic to an aqueous phase, and (3) completed by the functionalization of the nanocapsules with interleukin-2.

In the following, these three main steps will be assessed stepwise to allow detailed control and therefore good reproducibility of the entire complex procedure.

### Step 1: HES Nanocapsule Formation in Inverse Miniemulsion

**Figure 1:**
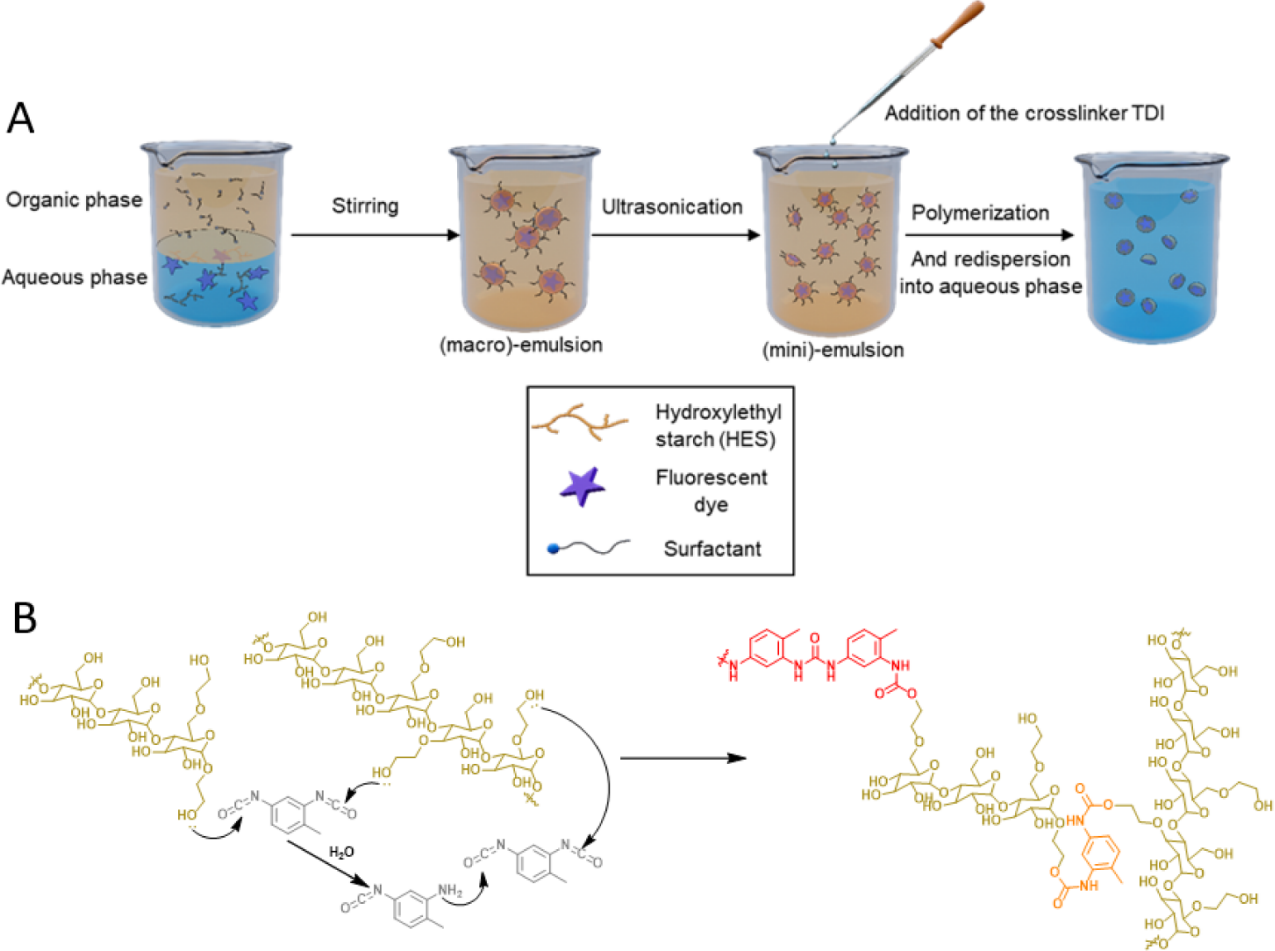
(A) Schematic procedure of hydroxyethyl starch nanocapsule (HES-NC) synthesis by inverse miniemulsion. (B) Chemical reaction scheme for the synthesis of HES-NCs. HES (yellow ocher) reacts with TDI (gray) forming a polyurethane polymer (orange), or in a side reaction, hydrolyzed TDI reacts with HES forming urea groups (red).

In the first step, hydroxyethyl starch (HES) nanocapsules (NCs) were synthesized by an interfacial polymerization reaction in inverse miniemulsion. Therefore, HES was added into the aqueous dispersed phase and reacted with toluene diisocyanate (TDI) in a polyaddition at the droplet interface, generating NCs (Figure 1). To illustrate the process dependency of the nanocapsule synthesis, the parameters are listed in Table 1.

**Table 1:** Process and Control Parameters of Nanocapsule Synthesis

Process parameter	Control/characterization parameter
Reproducible homogenization	Droplet size
Polymerization	Amount of cross-linker
Colloidal stability	Amount of surfactant
Labeling	Encapsulation efficiency
Surface functionalization	Surface analytics/assays
Biological effectiveness	Cell uptake/cell growth/bioassays

Furthermore, one of the requirements is the use of identical chemicals from the same supplier (and with the same potential impurities) to achieve the same reaction conditions. Therefore, the chemicals used are given in Table 2, and the necessary specifications are listed.

**Table 2:** Used Chemicals and Specifications for the Synthesis of HES-NCs

Chemical	Specifications
HES solution (3%)	Sterile from infusion bag
NaCl	Osmotic agent
SR101 or Cy5 Oligo	Fluorescent marker: Cy5 Oligo in RNase-free water
Cyclohexane	Dried (molecular sieve 5 Å)
Poly((ethylene-cobutylene)-*b*-(ethylene oxide) (P(E/B)-*b*-EO))	Synthesized with a molecular weight of *M*_w_= 8500 g/mol (with 6800 g/mol of the P(E/B) block and 1700 g/mol PEO) determined with GPC
Toluene diisocyanate (TDI)	Stored at –20 °C and freshly dissolved in cyclohexane

#### Reproducibility of the Capsule Size

The capsule size is one of the most crucial parameters for a successful and applicable synthesis. A stable size distribution from batch to batch is an important indication that capsule synthesis is reproducible because this means that droplet formation in miniemulsion is underlying a repeatable mechanism exhibiting optimal droplet stabilization. An important point to reduce batch-to-batch variance is the use of the same equipment for each synthesis. Especially, ultrasonic baths often have strong frequency fluctuations, and ultrasonic tips must not show any damage like scratches, which can lead to a change of the surface due to an unequal distribution of sonic waves.

For homogenization of the emulsion, an ultrasonic (US) tip or high-pressure microfluidics can be used, which could lead to different results in the size distribution of the HES-NCs. In addition, the attachment of different dyes either inside the capsule or outside the capsule can influence capsule formation.

**Figure 2:**
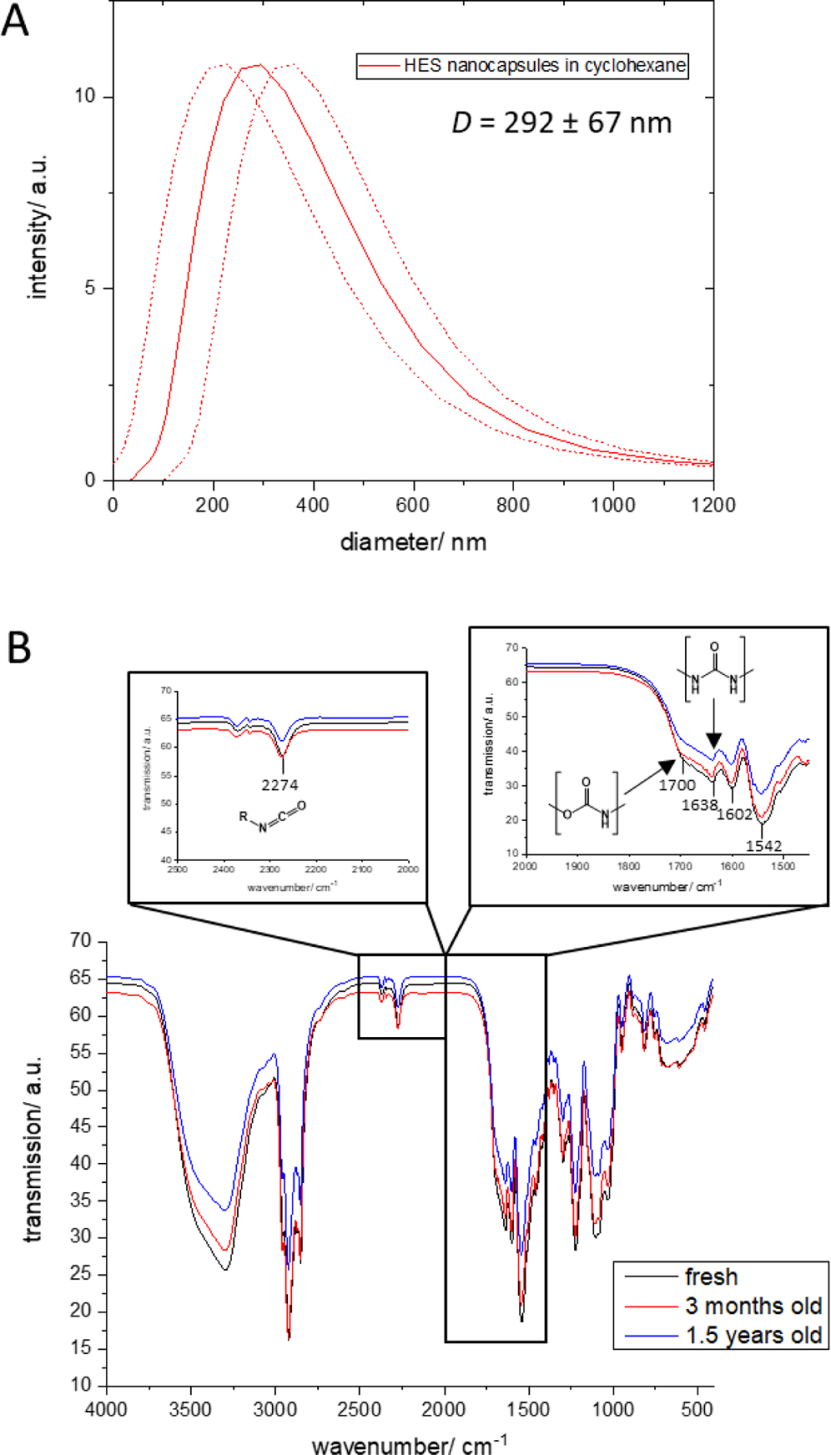
(A) Average diameters of 21 different HES-NC samples measured with DLS at a scattering angle of 90° (solid line). Dotted lines represent respective size distributions of the standard deviation of the averaged curve. (B) FTIR spectra of dried NC dispersions after storage in cyclohexane for different time periods.

The control of the size distribution from batch to batch is therefore very important to understand if droplet formation is constant and if stabilization is optimal. For this reason, the average diameter of the NCs was determined by DLS after their synthesis. Figure 2A shows the average diameter of 21 HES-NC batches, which were synthesized independently in time.

For data analysis, the intensity-weighted size distributions of the individual NCs samples were averaged (Figure 2A, solid curve), and the standard deviation of the obtained intensity maxima was determined. In this way, a value of 292 ± 67 nm was obtained as the nanocapsule diameter averaged over 21 different HES-NCs samples. The obtained result demonstrates that a simple 90° scattering setup can be used as a quality control to receive information if the synthesized NCs reveal comparable results and if the NC synthesis was conducted in a reproducible manner. Figure 2A reveals that the synthesis can be conducted in a time-independent and reproducible manner without large batch-to-batch variations.

#### Stability of the NC Dispersion in Cyclohexane

In a polyaddition reaction, the isocyanate groups of the TDI (Figure 1B, gray) react with nucleophiles such as the hydroxyl groups of HES (Figure 1B, yellow ocher). As a side reaction, TDI also reacts with amino groups, which are generated by hydrolysis of TDI in the aqueous emulsion forming polyurethane (Figure 1B orange) and polyurea (Figure 1B, red).

Polyurethanes, which are typically produced by a polyaddition using TDI, are known for their good stability and toughness.^23^ However, colloidal materials such as HES-NCs can have other properties, since during the miniemulsion, also urea groups are formed. For this reason, it is important to assess the conversion of TDI during the reaction and therefore evaluate the chemical stability of the NCs in order to determine the time of further processing.

To evaluate the chemical stability of the generated HES-NCs, three different HES-NC dispersions in cyclohexane of different ages were dried and measured by FTIR spectroscopy (Figure 2B).

In all the spectra, the characteristic vibrations for carbonyl containing compounds were found. The band at 1638 cm^–1^ (C=O stretching, secondary amide, see inlet) can be attributed to the polyurea vibration and the band at 1700 cm^–1^ (C=O stretching, see inlet) to the polyurethane vibration.^24^ In all spectra, the characteristic isocyanate band is visible (*v* = 2274 cm^–1^, *N* = C=O stretching). The appearance of the isocyanate band in all dispersions illustrates that TDI was not fully converted to urethane and urea groups during the polyaddition and was also not further converted in the cyclohexane phase during the storage time.

By means of FTIR measurements, we show that the dispersions remain chemically stable in organic solvent over 1.5 years, which is fundamental for the further processing of the NCs, since they can be stored over this period of time without chemical alterations, such as further hydrolysis of TDI.

However, usually in an aqueous environment, a change is detected. As soon as the NCs are transferred to water, an exchange between the inside of the capsule and the external environment occurs. For example, diffusion processes can lead to the release of small encapsulated water-soluble molecules, leading to different concentrations of the cargo or different salt concentrations changing the osmotic pressure inside the capsules. Furthermore, in water, biopolymers, such as HES, are able to incorporate water molecules leading to swelling and therefore to size alterations.^25^ For this reason, HES-NCs should always be stored in the organic phase before further processing.

### Step 2: Redispersion of HES-NCs in Water

After synthesis via inverse miniemulsion, HES-NCs are redispersed in water containing a water-soluble surfactant. Then, functionalization with biomolecules and application in biological media is possible. Redispersion is a critical point for reproducibility since the formed NCs are transferred to water exhibiting a different density. Especially, biopolymers show a different behavior in organic solvent than in water causing them to incorporate water molecules which leads to swelling of the material.^26^ Furthermore, ultrasonic baths often have strong fluctuations, and therefore, keeping reaction conditions constant is essential to obtain reproducible results for each redispersion.

**Figure 3:**
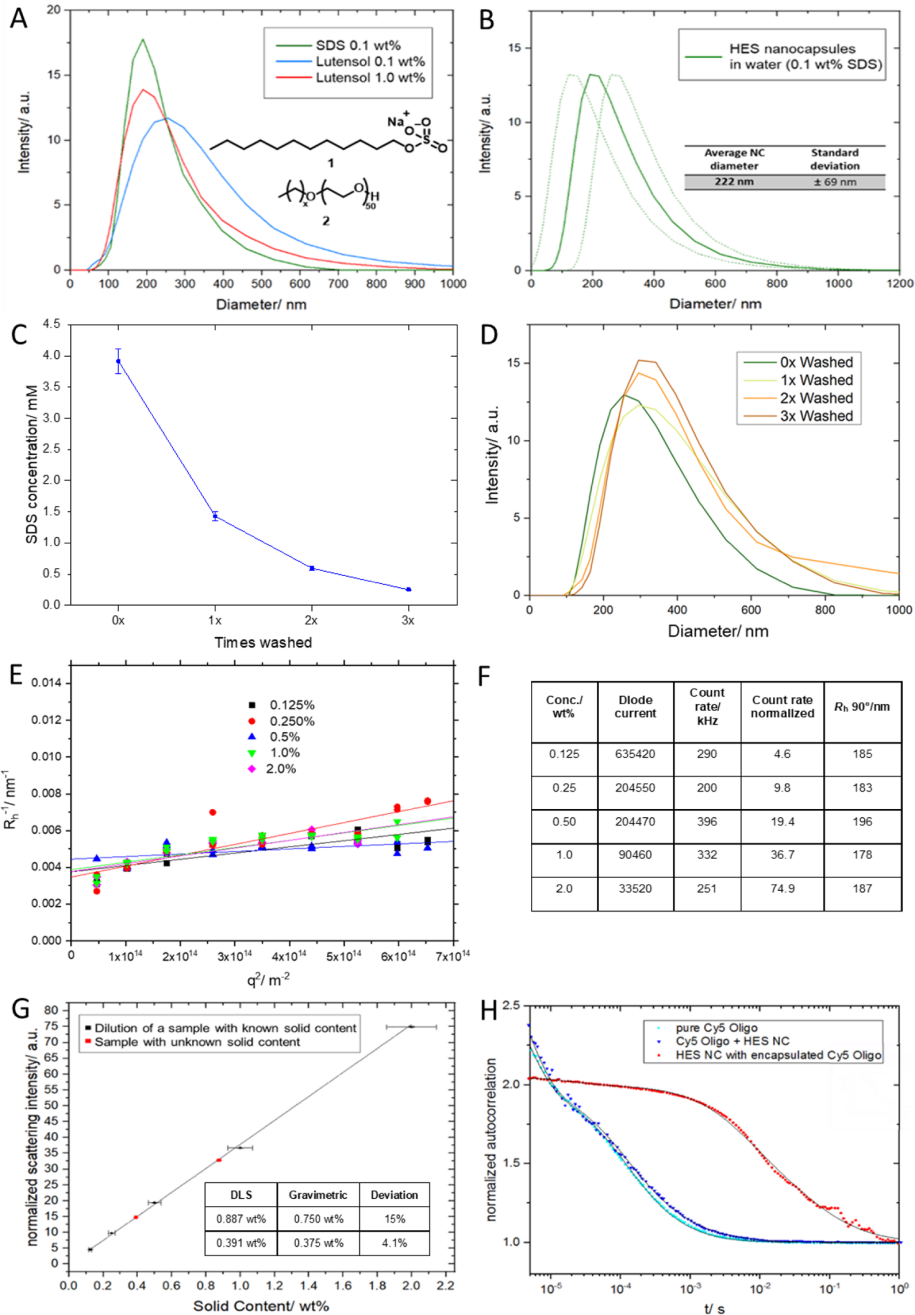
(A) Size distribution of redispersed HES-NCs in water using either SDS (**1**) or Lutensol AT50 (**2**). (B) Average diameter of HES-NCs stabilized with 0.1 wt % SDS after 28 different independent redispersion steps at a scattering angle of 90°. (C) SDS concentration in HES-NC dispersions decreases with the amount of washing. (D) Size distribution at a scattering angle of 90° of an HES-NC dispersion after washing cycles. (E) Inverse of the hydrodynamic radius obtained from dynamic multiangle light scattering (DLS) plotted against scattering vector *q*^2^ together with a linear extrapolation *q*^2^ → 0. (F) Scattering intensities and respective concentrations of an HES-NC dispersion were obtained from DLS at a scattering angle of 90°. (G) Relation between the solid content of an NC dispersion with the normalized scattering intensity during LS measurements (black data points represent values from F, calibration with known concentrations) and test of a second independent sample (red data points) for comparison with the gravimetrically determined solid content (see inserted table). (H) Fluorescence correlation spectroscopy of HES nanocapsules with Cy5 Oligo encapsulated (red) or with free Cy5 Oligo (cyan and blue) measured in water.

For adequate stabilization of the formed NCs in water, a surfactant needs to be added. It was reported that sodium dodecyl sulfate (SDS, Figure 3) is a suitable surfactant for HES-NC redispersion.^27−29^ Therefore, the NCs were redispersed in 0.1 wt % sodium dodecyl sulfate (SDS), and 28 different batches, which were again prepared independently of each other, were investigated using DLS. Figure 3B shows the average diameters of these NCs. Furthermore, the ionic surfactant SDS was compared with the nonionic surfactant Lutensol AT50 (Figure 3A).

The ionic SDS as well as nonionic Lutensol AT50 is able to stabilize redispersed HES-NCs in the aqueous continuous phase (Figure 3A). Both surfactants show size distributions comparable to those after synthesis in cyclohexane (compare Figure 2A). For SDS, it is sufficient to use 0.1 wt % for a proper stabilization, while the same stabilization effect can be achieved using 1.0 wt % Lutensol AT50 (Figure 3A, red curve). If a Lutensol concentration of only 0.1 wt % is used, it results in a broader size distribution and higher average size (Figure 3A, blue curve).

Based on this result, it can be concluded that ionic stabilization of the NCs in water has a more advantageous effect than steric stabilization during water transfer. This is in contrast to the stabilization of the NCs in cyclohexane, where steric stabilization by the surfactant P(E/B)-*b*-EO) is achieved. Further information about adequate stabilization can be obtained by the critical micelle concentration (CMC) of the surfactant. The CMC of SDS at 25 °C is 8.2 mM (0.24 wt %), while the CMC of Lutensol AT50 is 2 × 10^–3^ mM (4.92 × 10^–4^ wt %).^30,31^ It was reported that SDS is a very efficient colloidal stabilizer, while nonionic surfactants have a different stabilizing mechanism, resulting in poorer nucleating capacities.^32,33^ For this reason, in the case of nonionic surfactants, stabilizing effects improve even using concentrations above the CMC.^26^

Since the efficient stabilizing effect of SDS was demonstrated, the reproducibility of the redispersion process was studied using 0.1 wt % of SDS as surfactant.

Figure 3B shows the size distribution of 28 HES-NC samples in an aqueous SDS solution. These aqueous dispersions are redispersed samples originating from different HES-NC batches in cyclohexane. Again, for data analysis, the intensity-weighted size distributions of the individual NCs samples were averaged (Figure 3B, solid curve) to obtain a size distribution of all samples. From this curve, the averaged NC diameter (222 ± 69 nm) was obtained, revealing a slightly smaller capsule diameter than in cyclohexane. From the low average NC diameter and the comparable standard deviation as in the step before, a high reproducibility of the redispersion process can be concluded. The standard deviation of 69 nm, which was calculated as in the section above comparing the maximum intensity of the 28 distribution curves, reveals just a slightly higher value than that for NCs in the cyclohexane phase.

The slightly increased standard deviation can be explained by a higher variance of the individual size distributions of water samples. These results indicate that the redispersion process causes a higher variation of sample size distributions than that right after synthesis. However, this was expected as the samples originated from different cyclohexane batches, exhibiting differences in size distributions among themselves.

For biological testing, the SDS content has to be decreased to a minimum as it is toxic to cells since it was reported to induce significant conformational alterations to proteins such as lysozyme at concentrations of 0.2 mM.^34^

SDS concentrations should be kept as low and constant as possible to ensure reproducibility and biocompatibility of the NC samples. SDS concentrations as low as approximately 0.1 mM can be easily assessed using a colorimetric assay for SDS detection. In the assay described by Rusconi et al., it is shown how the carbocyanine dye stains-all can be used to quantify SDS levels in the microgram range, for example, in protein samples, since proteins can be very sensitive to denaturation by SDS.^35^ In our studies, we show that this protocol can also be applied to HES-NC dispersions to determine their SDS content. The absorbance spectra of the stains-all dye show an absorbance maximum at 510 nm in the absence of SDS and at 453 nm for SDS concentrations up to 10 mM (Figure S1). However, the best linearity of the measured absorbance was found at 438 nm so that this wavelength was chosen for measurement instead of 453 nm. Absorbance spectra of stains-all in the presence of HES-NCs are shown in Figure S2.

In our studies, we showed that the SDS on the NCs was effectively removed by means of centrifugal washing steps. Since the SDS concentration is proportional to the measured absorbance of the dye, an external calibration was performed. After each washing step, the amount of SDS present in dispersion was determined (Figure 3C) and the resulting capsule sizes were analyzed (Figure 3D).

The SDS concentration decreased rapidly after each centrifugal washing step and was reduced up to 10-fold of the originally added amount by three washing steps (Figure 3C). After these steps, SDS does not play a significant role for cytotoxicity anymore since the detected SDS amounts are very low, and in addition, more centrifugal washing steps increase the risk for aggregation of the capsules. The size distribution of the washed dispersions changed marginally during this procedure (Figure 3D). To obtain these results, washing was performed carefully, which means rather slower centrifugation speed but longer spinning time.

The quantification of SDS in an NC dispersion and simultaneous check for possible size alterations, e.g., due to changes in aggregation behavior, is an important quality control. SDS concentrations should be kept as low and as constant as possible to ensure the reproducibility and biocompatibility of the NC samples.

#### Yield of the NC Dispersion: The Solid Content

In contrast to molecules, for which there are numerous methods for the determination of their concentration such as various chromatography techniques and UV–vis or NMR methods, there is only a limited number of possibilities for colloids. If the NCs are completely insoluble in different kinds of solvents, only a gravimetric determination of the concentration is possible. The exact gravimetric determination of the solid content of the dispersion is difficult to perform, as possible surfactant residues and salts are also measured during weighing. Furthermore, it also leads to a high loss of material since NCs cannot be recovered after drying. Dynamic light scattering not only allows one to determine the size of NCs but also can be used to analyze the solid content of the sample. In principle, this is possible as the intensity of scattered light depends linearly on the concentration of scattering objects. This means that in dispersions with a very narrow size distribution, the scattering intensity is proportional to the solid content. However, it needs to be noted that this is only valid if the underlying assumption is correct that also the refractive index of the material stays constant, which means that the material should have no heterogeneities in composition. Prior to the analysis of the solid content of an NC sample by DLS, calibration samples were gravimetrically analyzed from a dilution series from 2 wt % down to 0.125 wt %. Then, dynamic light scattering was performed with these differently diluted HES-NC dispersions and besides the hydrodynamic radius, the diode current and the count rate were obtained (Figure 3E/F).

Since the diode current, which basically represents the laser intensity, cannot be kept constant during measurement for all different concentrations, the count rate is normalized to the respective diode current (count rate normalized).

The measured hydrodynamic radii of the samples differ from 178 to 196 nm, revealing reproducible capsule sizes within the dilution series. The values obtained (see Figure 3F) were used as standard calibration, whereby the scattering intensity of the samples was determined by DLS and plotted against the gravimetrically determined solid content (Figure 3G, black data points). Then, the solid content of another HES-NC sample was first determined gravimetrically, and afterward, light scattering measurements were performed, using the standard calibration to determine the solid content by the new method (Figure 3G, red data points). The results of both methods show high consistencies in the determination, especially at low solid contents.

In Figure 3G, a linear correlation between the normalized scattering intensities of the dilution series and the solid content was obtained, indicating that light scattering can be used for the determination of the concentration of HES-NCs dispersions (black data points). The solid contents of two dilutions of a second sample revealed that the obtained results from both methods are very similar with regard to both measurement inaccuracies ((1) DLS: 0.887 wt %, gravimetry 0.750 wt %, deviation: 15%; (2) DLS: 0.391 wt %, gravimetry 0.375 wt %, deviation: 4.1%)). This means that the LS method of concentration determination via a calibration curve can be very useful to perform fast quality controls of the same batch, e.g., during upscaling or when only very limited sample amounts are available. It needs to be pointed out that for a meaningful determination of the solid content, the hydrodynamic radius and the polydispersity of the sample must be constant within the error range, as the size and the size distribution influence the scattering intensity even more than the concentration.

#### Detectability of Encapsulated Markers

To detect the NCs in a biological surrounding like, e.g., after uptake by a cell, a fluorescent dye was encapsulated. Therefore, it is important that the dye to be detected must remain colocalized with the NCs. For this reason, fluorescence correlation spectroscopy (FCS) was used to investigate whether an oligonucleotide dye was encapsulated in HES-NCs after synthesis or was adsorbed to the outer surface of the capsule. The oligonucleotide dye Cy5 Oligo was chosen as it has a size of about 5 kDa, which is sufficient to prevent it from diffusing out of the NCs. Under these conditions, FCS was first performed with the pure dye (data points in cyan) and then with HES-NCs without dye to which Cy5 Oligo was added (blue data points). The data points in red show HES-NCs where Cy5 Oligo was added to the dispersed phase of the emulsion and where it was expected that the dye would be encapsulated after polymerization (Figure 3H).

FCS reveals that the oligonucleotide dye Cy5 Oligo remained encapsulated in HES-NCs and that there was no interaction between the capsule surface and freely diffusing Cy5 Oligo in the case that it was not encapsulated (blue curve). The measured fluctuations of fluorescence of free Cy5 Oligo dye (cyan) and unlabeled NCs with additionally added Cy5 Oligo (blue) show a similar behavior, while HES-NCs with encapsulated Cy5 Oligo reveal a different correlation function (red) (Figure 3H).

These findings demonstrate that there is no adsorption of free Cy5 Oligo dye to the capsule surface and additionally a clear colocalization of dye and NC, if the dye is encapsulated. Since the oligo dye is located inside the NCs when it is added during the emulsion (red curve), a free diffusion of the dye is prevented, and the detected diffusion coefficient correlates with the diffusion coefficient and thus the size of the nanocarriers. Due to the high water solubility and the large molecular size of the Cy5 Oligo dye, an encapsulation and therefore a colocalization of dye and NCs are observed. This property is important if the presence of nanocarriers in biological environments has to be verified, as is often done by fluorescence measurements. Additionally, the concentration of free dye (i.e., resulting from insufficient washing or leaking) can be quantified by FCS measurements and was approximately 3%, allowing for reliable detection of the HES-NCs.

### Step 3: Functionalization of HES-NCs

The cross-linker toluene diisocyanate (TDI) is sensitive to aqueous media hydrolyzing to amino groups. The FTIR spectra in Figure 2B provide evidence by showing typical polyurea vibrations, confirming the reaction of isocyanates with amines.^36^ In the following, ToF-SIMS experiments of capsule films were used as control parameters to verify amino species on the capsule surface. For the measurements, NC dispersions were applied as a multilayered film on a glass substrate, and the upper surfaces of these films were then analyzed, detecting secondary ions desorbed from the films as a result of primary ion bombardment.

**Figure 4:**
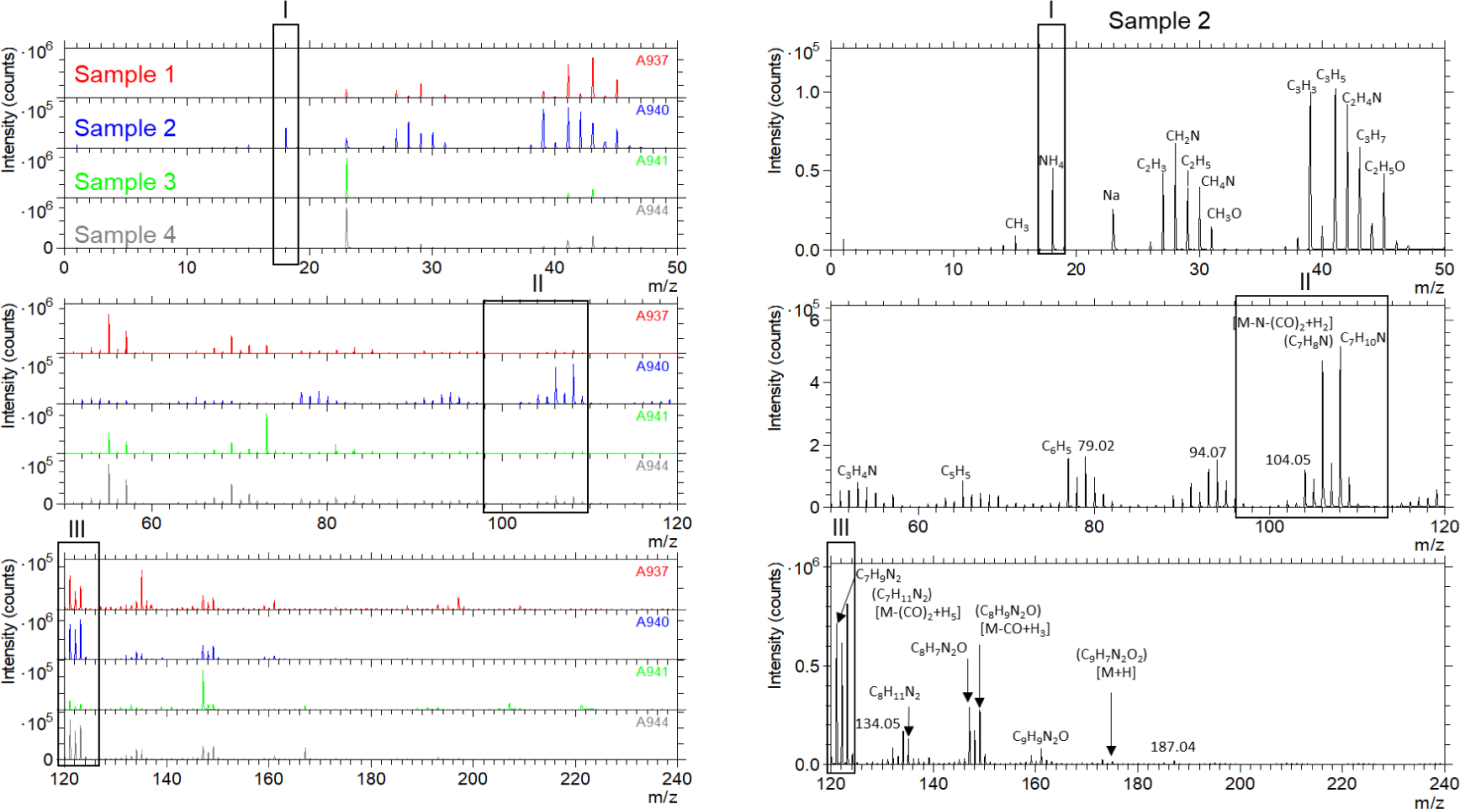
ToF-SIMS surface spectra of the prepared samples 1–4 in positive ion mode. Black boxes (I–III) mark prominent signals. On the right-hand side, prominent patterns of sample 2 are exemplarily shown which are also found in the other samples.

To investigate possible differences in the surface composition after the redispersion process, different samples were prepared and the dispersions were purified by centrifugation. Spin coating of the capsule dispersions on the glass substrate resulted in a colloidal film. Due to hydrolysis of residual TDI during the redispersion of HES-NC to water, it was expected that a significant increase of amino groups on HES-NCs in water (Figure 4, sample 1) compared to HES-NCs in cyclohexane (Figure 4, sample 2) occurs and will be detected in the films. Furthermore, two additional samples were prepared in which an aqueous HES solution and TDI were emulsified in cyclohexane without a surfactant and reacted overnight. The resulting polymer was dissolved in a mixture of DMSO and THF and applied to the substrate (Figure 4, sample 3). In the fourth sample, HES was dissolved in DMSO and applied to a glass substrate. Subsequently, TDI, dissolved in DMSO, was added to the HES solution and reacted in situ on the glass substrate with HES (Figure 4, sample 4). Using the prepared films 3 and 4 (samples 3 and 4), the reaction of HES and TDI in bulk should be compared with the reaction in emulsion. For film processing, the dispersions were spin-coated with a speed of 500 rpm on the glass substrate, and the polymer solutions were drop-casted and dried overnight. Afterward, the obtained films were analyzed.

ToF-SIMS analyses of the different films reveal several signals found in all four samples. In addition to a number of very prominent signals for the typically encountered hydrocarbon species (in the range of *m*/*z* 25–80), a signal for the ammonium ion NH_4_ (I) was found which revealed the strongest intensity in sample 2 and a weak intensity in sample 1. Signals at *m*/*z* 106.07 and 108.07 (II) demonstrate a notable pattern in sample 2, which are associated with the fragments C_7_H_8_N and C_7_H_10_N and can be attributed to hydrolysis products of TDI. Furthermore, intense signals at 121 to 123 *m*/*z* (III) were found in all samples which are attributed to the fragments C_7_H_9_N_2_ and C_7_H_11_N_2_. (Figure 4). The finding of these amino-containing fragments suggests the successive hydrolysis of the isocyanate TDI.

To control the binding of biomolecules, DBCO-PEG-NHS ester was attached to the capsule surface to exploit a copper-free click reaction on the capsule surface. This reaction is known to be very efficient, as it provides high yields at room temperature and in aqueous solutions.^37^ The absence of copper results in a better biocompatibility of the functionalized NCs as traces of the metal are toxic, and complete removal is difficult. Adjusting the concentration of the surface groups allows a specific binding of following azide-terminated biomolecules and therefore the awarding of biological functions to these HES-DBCO-NCs.

The DBCO on the capsule surface was quantified by a fluorescent assay using anthracene azide in an earlier published procedure and by means of an external calibration of the generated fluorescent triazole complex.^23^

**Figure 5:**
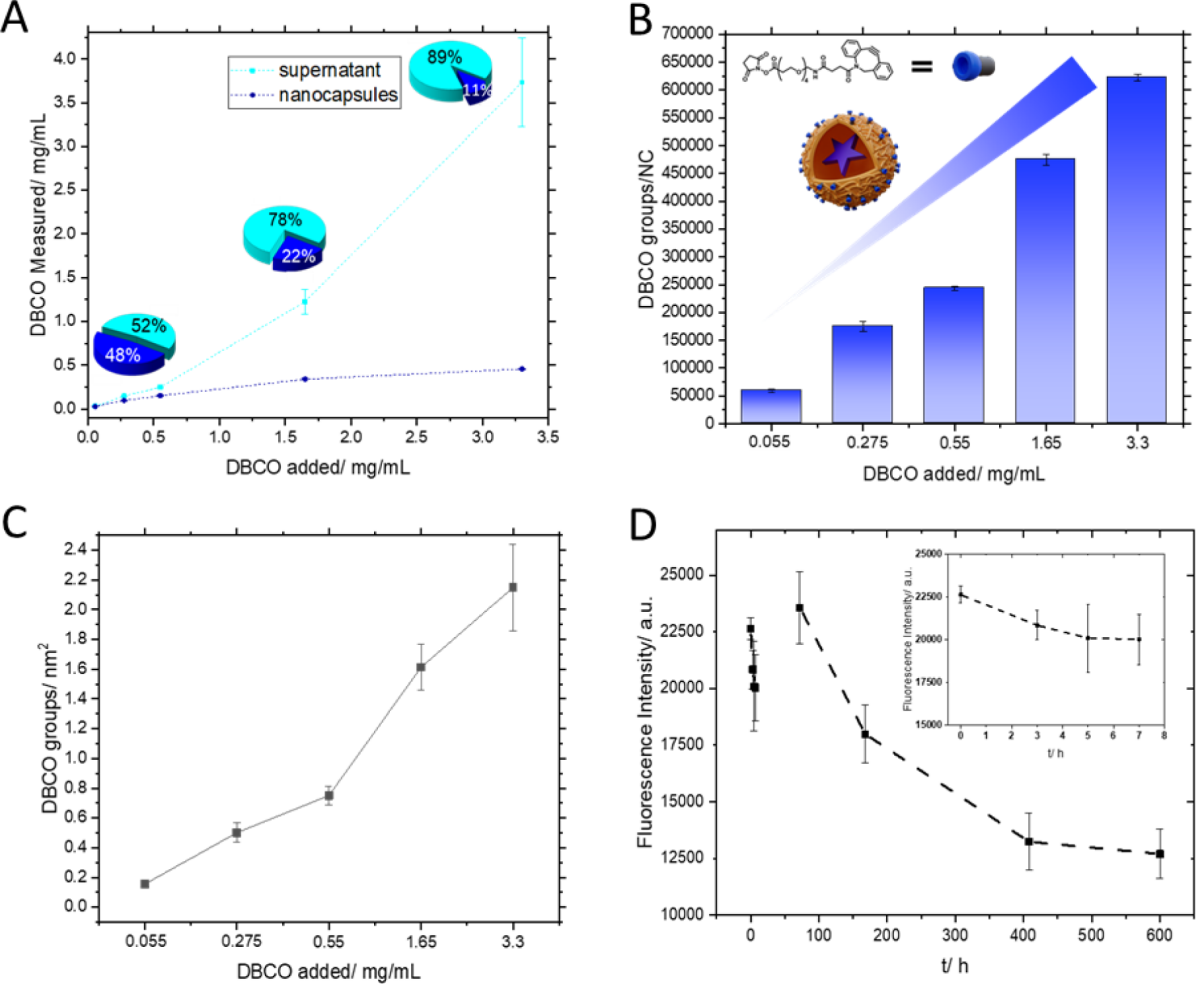
(A) Comparison of initially added and measured amounts of DBCO to the NC dispersion. The data points/pie charts in cyan represent the DBCO found in the supernatant, while the data points/pie charts in blue represent the DBCO found in the NC dispersion after the addition of different DBCO concentrations and subsequent washing. (B) Total amounts of DBCO on the NC surface are in groups/NC. (C) DBCO groups per nm^2^ were determined for different bound DBCO concentrations to HES-NCs. (D) Time-dependent decrease of the fluorescence activity of the formed complex of DBCO-PEG-NHS ester with anthracene azide.

The total amount of added DBCO ester was recovered in the sum of detected DBCO concentrations in the supernatants and in the dispersion using the standard calibration of the fluorescent complex for DBCO quantification (Figure 5A). Moreover, it was shown that it is possible to bind DBCO in different concentrations to the capsule. With this knowledge, the DBCO density on HES-NCs was determined for the different added DBCO ester concentrations if the size of the NCs is known (Figure 5B). Moreover, the surface density of DBCO groups on the capsule surface was analyzed (Figure 5D).

Using a standard calibration of the formed complex, the amount of DBCO on the NC surface in an aqueous dispersion was determined. At concentrations starting from around 0.5 mg/mL (4.635 × 10^17^ DBCO groups/mL) and higher, more DBCO was found in the supernatant than in the NC dispersion, which shows that a surface modification of the HES-NCs with DBCO-NHS ester at such concentrations does not lead to an efficient attachment anymore (Figure 5A). The linear increase of DBCO in the supernatant between a concentration of 1.6 and 0.5 mg/mL is not as steep as at higher concentrations, which could indicate that interactions between DBCO and the dispersion within this concentration window still occur. The curve for the DBCO amounts detected in the NC dispersion reveals a DBCO saturation of the HES-NCs dispersion at around 1.5 mg/mL of DBCO, since a constant level of DBCO is reached (Figure 5A, blue curve). The less DBCO was added to the dispersions, the more it was also found bound on the NCs and not free in supernatants (Figure 5A, pie charts), indicating binding of DBCO ester to the HES-NCs. Figure 5B reveals that there is an increasing number of attached DBCO groups per NCs depending on the addition of DBCO ester, revealing the adjustability of the DBCO concentration on the HES-NCs. Up to 600000 groups were thus detected on the capsule. The error bars refer to measured fluorescence intensities and are comparably low (deviation 1–5%). At an addition of 0.055 mg of DBCO per mL of dispersion, approximately 60000 groups per NC were detected. Between the addition of 1.65 mg DBCO/mL and 0.55 mg/mL, a stronger decrease in DBCO groups per capsule was detected than with the other values. This is surprising because in Figure 5A, the curve in this area tends to flatten out, and accordingly, the curve for the detected amounts of DBCO in the supernatant starts to rise. Since the capsule size changed slightly depending on the degree of modification, this difference in the concentration-dependent evaluations of Figure 5A,B becomes apparent here. For this reason, the detected DBCO groups per nm^2^ were analyzed and revealed, as expected, a concentration-dependent decrease of detected groups with reduced addition of DBCO ester. Figure 5C reveals that for the highest DBCO concentration added, (3.3 mg/mL) 2.2 ± 0.2 groups/nm^2^ were found. Accordingly, for lower added concentrations, lower surface loadings were found. Depicted error bars were given as the percentage error of the sum of the error of the fluorescence measurement with the error of the size determination and are in the range between 8 and 13%. The results demonstrate a high surface coverage of the capsules by DBCO at added DBCO concentrations of 3.3 mg/mL with adjustability to a surface coverage to approximately 0.1 DBCO groups/nm^2^.

#### Stability of Added DBCO Groups

To investigate the storage stability of the DBCO groups and to gain insight into how fast the subsequent synthesis steps must be performed, the fluorescence intensity of the added DBCO ester in aqueous solution was investigated. Therefore, a time-dependent measurement of the fluorescence intensity was performed after addition of anthracene azide to DBCO. The DBCO ester was constantly stored in an aqueous solution, as it is the case for the DBCO molecules binding to the aqueous HES-NC dispersion, and freshly dissolved anthracene azide was added to DBCO samples, and the fluorescence intensity was checked subsequently.

The DBCO ester reveals a stable fluorescence intensity upon reaction with anthracene azide after storage in an aqueous solution for 7 h. A decrease in fluorescence intensity was visible after 3 days (Figure 5D), indicating a reduction in reactivity of DBCO, which might occur due to the high reactivity of DBCO and its reported sensitivity toward nucleophiles.^38^ This shows that after 3 days, a click reaction can still be efficiently performed using DBCO after it was attached to the HES-NCs. These findings suggest that the reproducibility of the click reaction is not affected when this time frame is complied with.

As well as pure DBCO ester, the fluorescence intensity of the formed triazole decreases over time in an aqueous medium (Figure S3). This decrease in fluorescence intensity of the triazole is attributed to a reduction in the excitation of the fluorophore and not to a chemical instability of the complex, since 1,2,3-triazoles are extremely stable and the 1,3-cycloaddition is irreversible.

#### Binding of IL-2 to HES-DBCO NCs

After the successful attachment of DBCO to the NCs, azidated biomolecules were clicked onto the NCs by copper-free click chemistry. Furthermore, proper functioning of the biomolecule after modification must be ensured.

**Figure 6:**
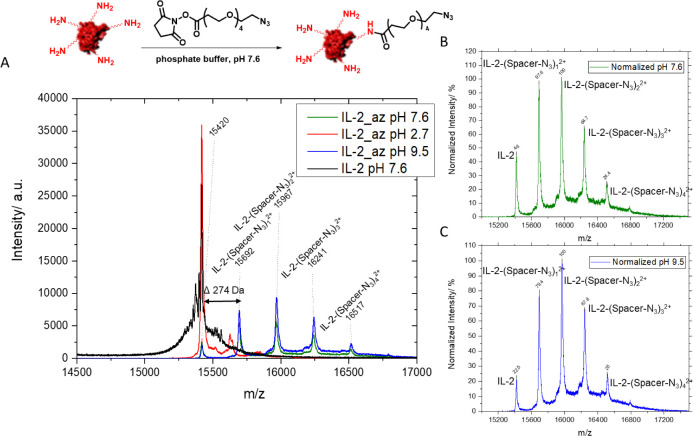
MALDI-TOF spectra of azidated IL-2 by NHS-PEG-azide 12 using different pH values (α-cyano-4-hydroxy cinnamic acid was used as a matrix). (A) Mass spectra overlay different conditions. The black curve shows unmodified IL-2 (15420 Da), while the other curves (green, red, and blue) show azidated IL-2 which reacted using three different pH values. The shown mass-to-charge ratios depict the different numbers of linker molecules attached to the protein. (B) Normalized relative intensity of mass patterns after the functionalization of IL-2 at pH 7.6. (C) Normalized relative intensities of mass patterns after functionalization of IL-2 at pH 9.5.

Using the cytokine IL-2, T cells can specifically be addressed, and their growth and differentiation can be stimulated.^1,2,4^ For this reason, it is important to attach IL-2 to a suitable nanocarrier to target specific T cell subpopulations.^4^ In the following, we show how IL-2 was attached to the HES-NCs. In the first step, IL-2 was modified with azide groups by NHS chemistry. By attaching the azide terminated linker (Figure 6) containing an NHS ester, nucleophilic lysines, which predominantly point to the outside of the protein, were addressed. The attached azide groups on the protein could be detected by MALDI-ToF, and it was shown that up to three linker molecules could be attached to the surface of IL-2 (Figure 6). Especially, MALDI-ToF is suitable for the detection of proteins and offers versatile application for all kinds of biomolecules.^39^

The mass spectrum highlighted in black was obtained from the IL-2 reference sample prepared in phosphate buffer at pH 7.6 and reveals a mass of 15420 Da which agrees with the values given by the supplier and by the literature (15.5 kDa).^40^ The observed pattern of 274 Da between the signals (Figure 6A, blue and green) corresponds to the mass of a single linker molecule. These findings demonstrate that the linker attached to the protein up to 4 times at neutral (green curve) and basic pH (blue curve). At pH 2.7 (Figure 6A, red curve), this pattern was not observed, indicating no attachment of the linker to the protein, since at acidic pH, the NHS ester did not react with the protein, due to charged lysine groups. Comparing the intensities of the signals of modified IL-2 samples (blue and green), we conclude that although there is a 3-fold molar excess of the NHS ester in the reaction mixture, there is unmodified IL-2 present. To compare the obtained signal intensities given for the modified protein in the mass spectra, the detector intensity was normalized to 100% for the most intense signal (Figure 6B,C). In both mass spectra, the most intense signal corresponded to the IL-2 sample with two bound azide linkers. However, the mass patterns reveal minor differences in the composition of modified IL-2 for different pH values.

If the relative intensities of the individual signals are considered in the sum of the total intensity, a balanced ratio of about 30% for the mono- and double-modified IL-2 was obtained for the reaction at pH 7.6 (Figure 6B). In contrast, a slightly higher proportion of the double-modified protein was obtained at pH 9.5 (Figure 6C). The proportion of 3-fold-modified IL-2 also appeared to be slightly higher at pH 9.5 than at pH 7.6, but in return, the proportion of unmodified protein was more significant at neutral pH than in the basic range.

For the 4-fold-modified variant, similarly low percentages were found for both pH values. The MALDI-ToF experiments demonstrate that IL-2 can be modified with the NHS-linker at pH 7.6 and pH 9.5, leading to a different composition of single and multiple modified protein. Hence, the results indicate that adjustment of the pH value is important for reproducible attachment of linkers to the protein and consequently also for reproducible binding to the NCs.

**Figure 7:**
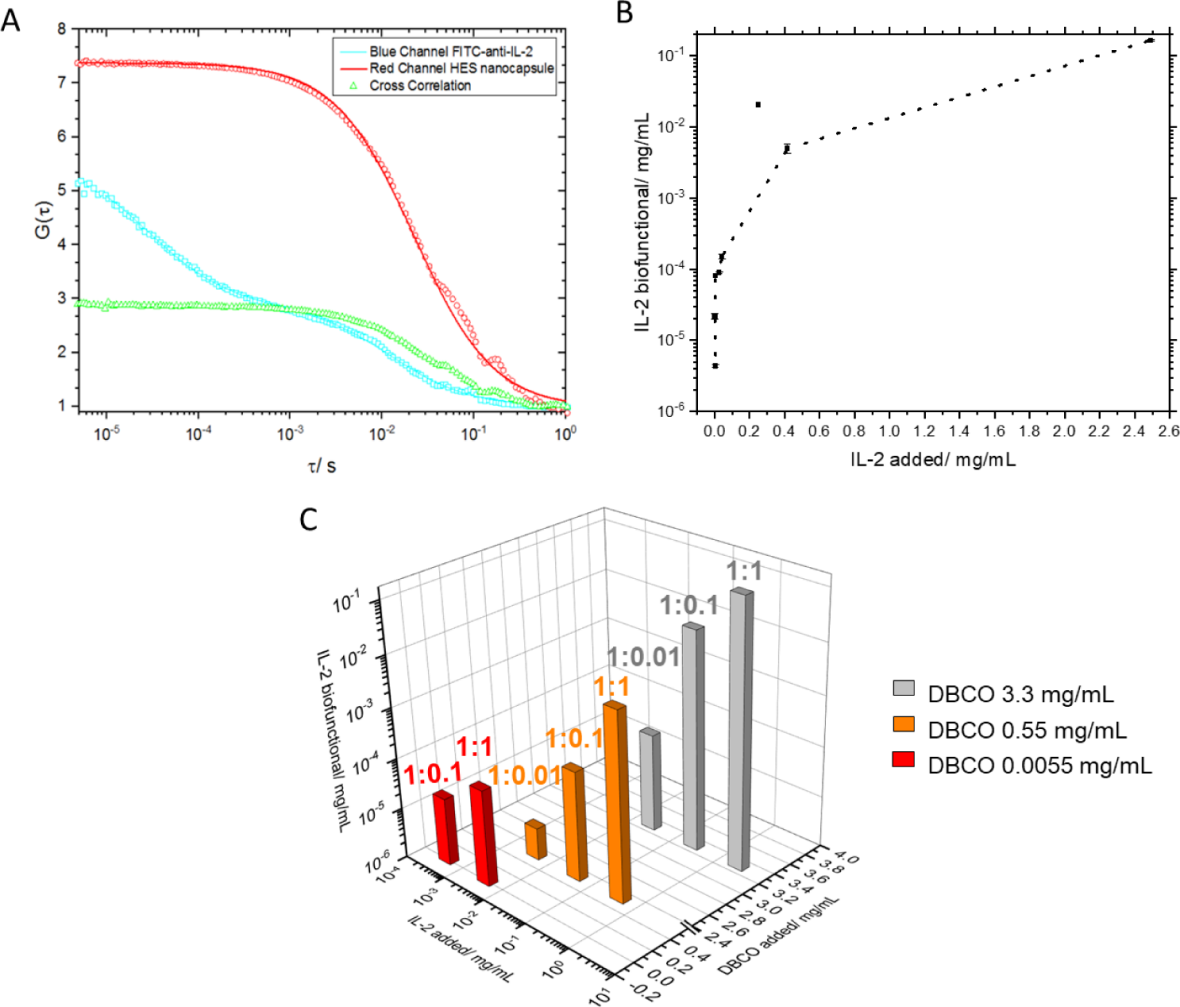
(A) FCCS measurement of IL-2-decorated HES NCs, with encapsulated Cy5 Oligo and with bound FITC-labeled anti-IL-2 antibody. The graph shows the fluorescence intensity of encapsulated Cy5 Oligo (red curve), the FITC-labeled anti-IL-2 antibody (cyan curve), and the cross correlation (green curve). (B) Correlation between added IL-2 to HES NCs and biofunctional IL-2 after attachment was analyzed by sandwich ELISA. (C) Dependency of the biologically active IL-2 on added DBCO concentrations and added IL-2 concentrations.

After modification of IL-2, it was subjected to reaction with the functionalized HES-NCs by a copper-free azide alkyne click reaction. To investigate the amount of covalently attached IL-2 on the NC surface, modified NCs (1 wt %) were incubated with FITC-labeled anti-IL-2 antibody and subjected to FCCS measurements (Figure 7A). Please note that “real” biofunction can be only analyzed by, e.g., functional T cell assays. However, a quantitative comparison of the amount of attached IL-2 and biofunctional IL-2 that can still be recognized by a cytokine specific antibody was analyzed by a sandwich-ELISA (enzyme-linked immune-sorbent assay) procedure (Figure 7B/C). Furthermore, this was compared to the number of DBCO groups attached to the surface (Figure 7C).

For the FCCS experiment (Figure 7A), the DBCO-modified HES-NCs were fully covered with azidated IL-2 in a 1:1 ratio, which means that for every DBCO, one IL-2 was added. The red curve represents the red fluorescence of the encapsulated Cy5 Oligo inside the HES-NC and the cyan curve the fluorescence of the bound FITC-labeled anti-IL-2 antibody on the NC surface. The green curve shows the cross correlation of the complex, which represents both fluorescent species moving together, demonstrating the binding of the components to each other. The curves reveal that there is still free anti-IL-2 antibody available, since the green curve does not lie perfectly between the red and the blue curve but approximates the red curve starting at lag times from 10^–2^ s. Therefore, it can be assumed that some of the anti-IL-2 antibodies bind to the IL-2-decorated HES-NCs, and these findings qualitatively prove the presence of IL-2 on the HES-NC surface.

Furthermore, a quantitative analysis of the amount of functional bound IL-2 was performed using a sandwich ELISA. In this method, IL-2 bound to NCs was detected with an anti-IL-2 antibody, which was fixed on a well plate. Figure 7B shows the correlation of added IL-2 to the NC dispersion to the concentration of the detected one with ELISA (referred to as biofunctional IL-2). If the added IL-2 concentrations are plotted in ascending order against the biologically active concentrations detected in ELISA, a logarithmic progression is observed. The flattening curve at higher concentrations indicates an approximation to a limit of bound IL-2. The concentrations detected by ELISA are several orders of magnitude lower than the added concentrations. This can be explained by the sterically limited accessibility and flexibility of the fixed anti-IL-2 antibody binding to the IL-2-decorated NCs (Figure 7B). The dependency between added IL-2 and biologically active IL-2 is additionally compared with a third dimension, that of the DBCO concentration used (Figure 7C). The three differently colored columns show three DBCO concentrations, which were incubated with three different IL-2 ratios each. The graph demonstrates that with high DBCO concentrations and correspondingly high IL-2 concentrations, many biologically active IL-2 can be found.

If ten times lower ratios of IL-2 to DBCO (1:0.1) are added to capsules exhibiting different DBCO concentrations (high, 3.3 mg/mL, 2 DBCO/nm^2^, low, 0.55 mg/mL, 0.75 DBCO/nm^2^, and very low, 0.0055 mg/mL), the biologically active amount is reduced accordingly. However, a constant decrease of the IL-2 concentrations was observed at ratios of 1:1 and 1:0.1 but no longer at 1:0.01, where the amount found was less than expected and corresponds more to the logarithmic course shown in the curve in Figure 7B. At the lowest DBCO concentration used (red columns, 0.0055 mg/mL), the 1:100 ratio is no longer detectable in ELISA. Furthermore, the results point to the fact that high amounts of DBCO are not necessarily required to obtain HES-NC with sufficient amounts of biologically active IL-2. For example, a DBCO amount of 0.0055 mg/mL (100-times reduced compared to 0.55 mg/mL) with a 1:10 ratio of DBCO and IL-2 (red column, 1:10) showed a higher detected amount of biofunctional IL-2 than the 10-times increased DBCO concentration with a 1:100 ratio of IL-2 (orange column, 1:100) (Figure 7C). The results show the attachment of different IL-2 concentrations to the HES-NCs and reveal information about the binding nature and detectability of bound IL-2 using an anti-IL-2 antibody.

### Biological Experiments: Concentration-Dependent Stimulation of CTLL-2 Cells

We have shown that HES-D-IL-2 NCs were was dose-dependently taken up by murine and human T cells, resulting in efficient T cell proliferation as previously demonstrated by CLSM and flow cytometry.^4^ Accordingly, we observed relevant percentages of HES-D-IL-2 positive and proliferating T cells by flow cytometry analysis that declined with reduced surface-bound IL-2 concentrations (Figure S4).

**Figure 8:**
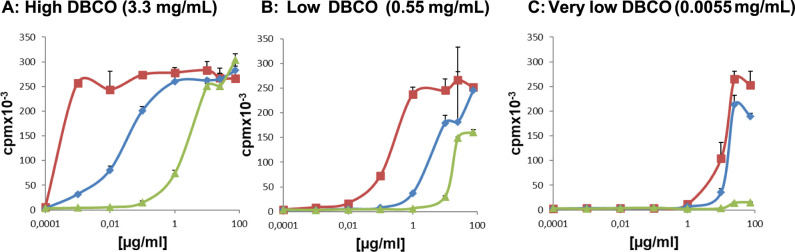
Counted radioactivity (counts per minute) of CTLL-2 cell proliferation plotted against added NC concentration in μg/mL. (A) The cell growth after incubation with NCs with high DBCO loading (3.3 mg/mL, 2 DBCO/nm^2^), (B) with low DBCO loading (0.55 mg/mL, 0.75 DBCO/nm^2^), and (C) with very low DBCO loading (0.0055 mg/mL). All graphs show the curve progression after incubation of CTLL-2 cells with the corresponding DBCO-modified NCs with three different IL-2 ratios calculated using the previously obtained DBCO concentration (red: HES-D-IL-2, green: HES-D-IL-2/10, and blue: HES-D-IL-2/100).

CTLL-2 cells, which are a murine T cell line, grow dose-dependently in response to the offered murine and human IL-2 concentrations. To evaluate whether the different surface loadings of DBCO and IL-2 have a biological impact, different HES-NCs were incubated with CTLL-2 cells. Figure 8 shows the growth of CTLL-2 cells dependent on bound IL-2 to the NC surface.

The proliferation of CTLL-2 cells is depicted by the counted radioactive decay, using a ^3^H-thymidine proliferation assay. The cells were incubated with HES-NCs modified with different surface loadings of DBCO (high, 3.3 mg/mL, low, 0.55 mg/mL, and very low, 0.0055 mg/mL) and corresponding IL-2 to DBCO ratios (1:1, 1:10, and 1:100, red, blue, and green curves in each graph) in different concentrations (Figure 8A–C). CTLL-2 cells proliferated dose-dependently on the presented IL-2 amount on the NCs (control measurements with pure IL-2 and unfunctionalized nanocapsules; see Figure S5). The cells which were incubated with NCs exhibiting the highest DBCO amount (3.3 mg/mL), and therefore also the highest IL-2 concentration attached (Figure 8A, HES-D-IL-2, red curve), showed the strongest proliferation at different added NC concentrations. If the DBCO amount on the NCs was kept constant, but the IL-2 amount was reduced, we observed a decreased proliferation curve (Figure 8A–C, HES-D-IL-2/10, HES-D-IL-2/100, blue and green curve). To obtain a high proliferative effect, as with high IL-2 loadings, more NC dispersions were used, to reach the maximum counts of 250 cpm. This level was found in all experimental settings with highest IL-2 loading (Figure 8, red curves and also with both IL-2 ratios at high DBCO loading) (Figure 8A, blue and green curve), as well as in the medium DBCO loading with the DBCO:IL-2 ratio of 1:10 (Figure 8B, blue curve). NC concentrations starting from 0.001 μg/mL (Figure 8A, red curve) up to 100 μg/mL (Figure 8A, green curve, B, blue curve, or C, red curve) were used to achieve a maximum proliferation of the cells.

These results indicate that selective and cytokine-concentration-dependent stimulation of T cells was achieved using different combinations of DBCO and DBCO to IL-2 ratios on HES-NCs. It is well-known that IL-2 stimulates different T cell populations depending on its concentration; e.g., high amounts of IL-2 result in activation of effector and memory T cells responses as useful in cancer therapy, whereas low levels of the cytokine lead to activation of regulatory T cells that control autoimmunity.^2,3^ Therefore, these findings are very important for development of nanocarrier-based immunotherapeutical approaches since by adjustment of IL-2 concentrations on the HES-NC surface, different levels of IL-2 modifications can be achieved, resulting in effective targeting of specific T cell subpopulations.

## Conclusion

We decorated the surface of HES nanocapsules with defined amounts of IL-2, so that different degrees of functionalization were achieved, thereby preserving the bioactivity of IL-2 and leading to the concentration-dependent growth of T cells. We demonstrate that by splitting up the synthesis into smaller steps and by controlling them, reproducibility of the synthesis is obtained, since individual changes can be followed more easily. Different DBCO concentrations present on the nanocapsules, and a loss of chemical stability results in a different surface loading of IL-2 and ultimately to a change in growth behavior of T cells. Our detailed protocol for the synthesis of a defined surface modification of nanocapsules by splitting up the synthesis in small steps displays a basic structure–activity relationship for a nanocarrier. We believe that such protocols can provide higher batch-to-batch consistency of nanocarrier systems and bring them one step closer to clinical application.

## Supporting Information Available

Reaction scheme of stains-all reaction with SDS and absorption spectra of dye SDS complex with different concentrations of SDS; absorbance spectra of dye SDS complex for washed HES NC and their supernatants after washing; time-dependent decrease of the fluorescence intensity of the formed DBCO-anthracene azide complex in water; flow cytometry measurements of human CD4^+^CD25^+^ T cells after incubation with 25 μg of high, low, and very low DBCO HES-D-IL-2; proliferation data of IL-2 dependent murine T cells (CTLL-2 cell line) (PDF)
