# Supplementary material for: Process Control of Multistep Surface Functionalization on Hydroxyethyl Starch Nanocapsules Determines the Reproducibility of the Biological Efficacy
Source: Biomacromolecules. 2024 Oct 22;25(11):7108–22. doi: 10.1021/acs.biomac.4c00490 (PMC11558556; doi:10.1021/acs.biomac.4c00490)
Supplement: Supplementary file 1 — bm4c00490_si_001.pdf [file bm4c00490_si_001.pdf]

Process control of multi-step surface functionalization on hydroxyethyl starch nanocapsules determines the reproducibility of the biological efficacy

Marie-Luise Frey[1], Svenja Morsbach[1], Matthias Domogalla[2], Volker Mailänder[2,1], Kerstin Steinbrink[3], Katharina Landfester[1,*]

[1] Max Planck Institute for Polymer Research, Ackermannweg 10, 55128 Mainz, Germany
[2] Dermatology Department, University Medicine of the Johannes Gutenberg-University Mainz, Langenbeckstr. 1, 55131 Mainz, Germany
[3] Department of Dermatology, University Hospital Münster, University of Münster, Von Esmarch-Strasse 58, 14948 Münster, Germany
* corresponding author, email: landfester@mpip-mainz.mpg.de

Additional Material

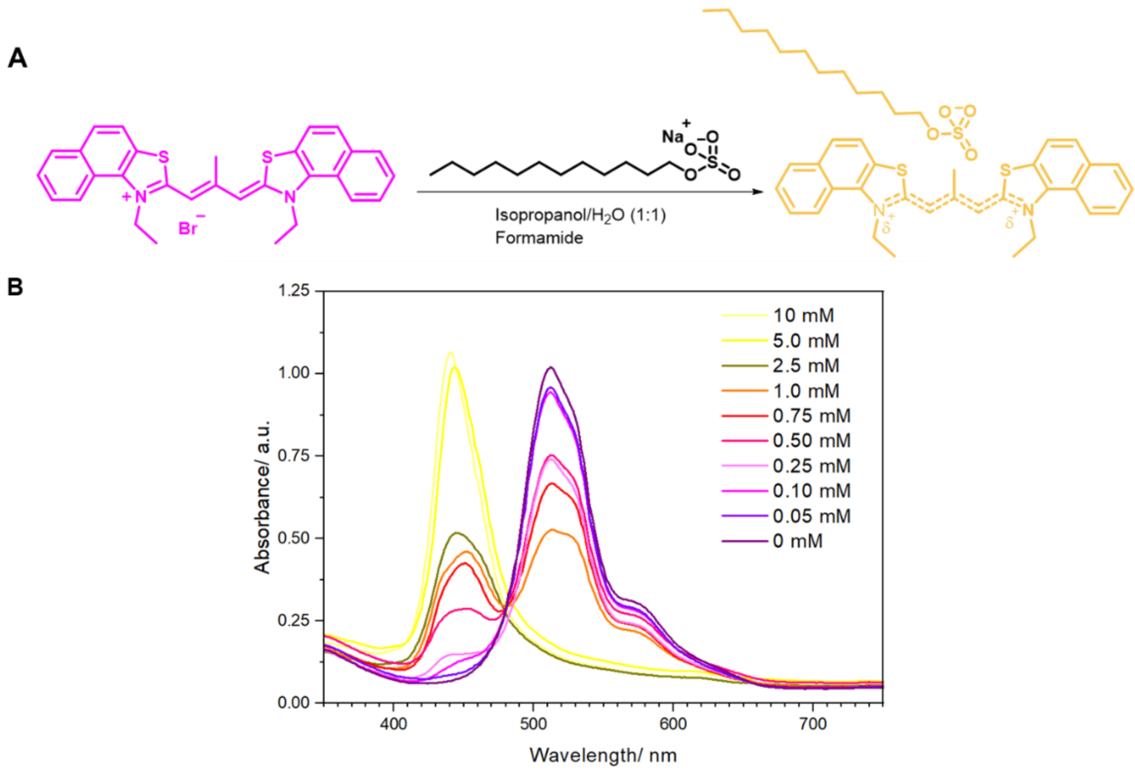

Figure 1: A) Reaction scheme of stains-all reaction with SDS. The purple stains-all turns yellow upon mixing with SDS. B) Absorption spectra of dye SDS complex with different concentrations of SDS.

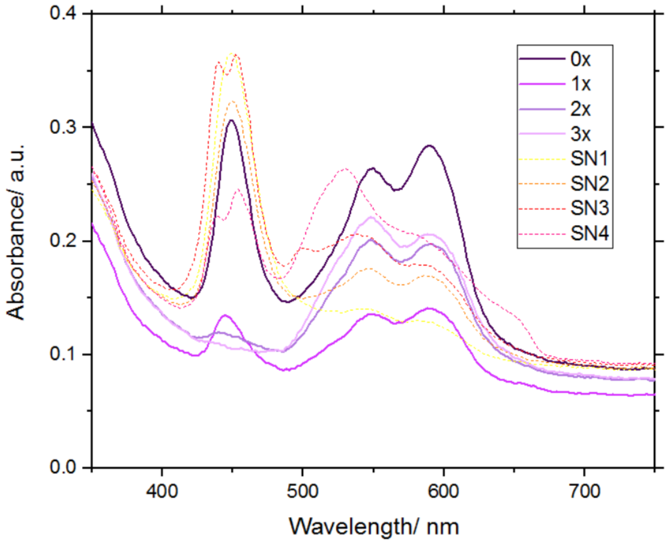

Figure 22: Absorbance spectra of dye SDS complex for washed HES NC and their supernatants after washing.

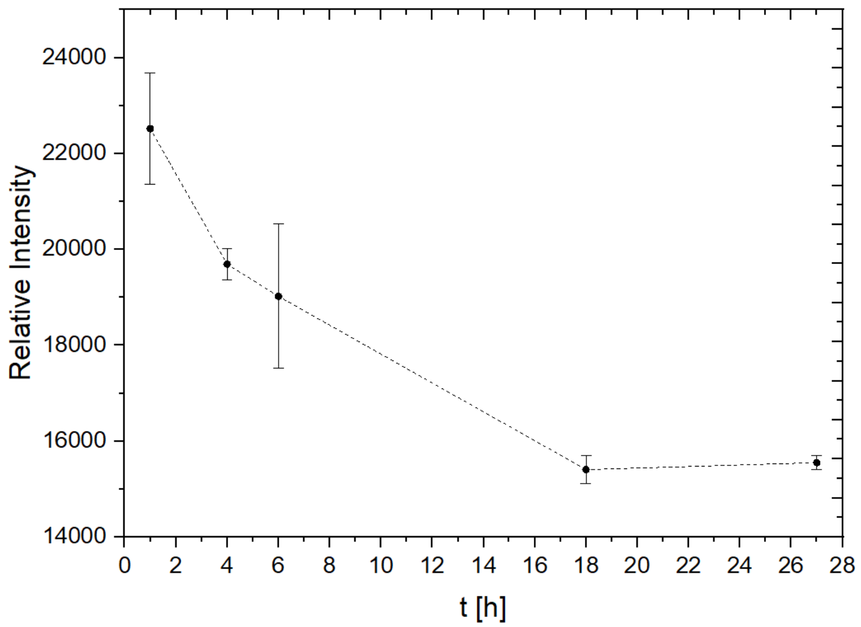

Figure 3: Time-dependent decrease of the fluorescence intensity of the formed DBCO-anthracene azide complex in water.

HES-D-IL-2 were dose-dependently taken up by murine and human T cells, resulting in efficient T cell proliferation as previously demonstrated by CLSM and flow cytometry.[1] Accordingly, we observed relevant percentages of HES-D-IL-2 positive and proliferating T cells by flow cytometry analysis that declined with reduced surface bound IL-2 concentrations.

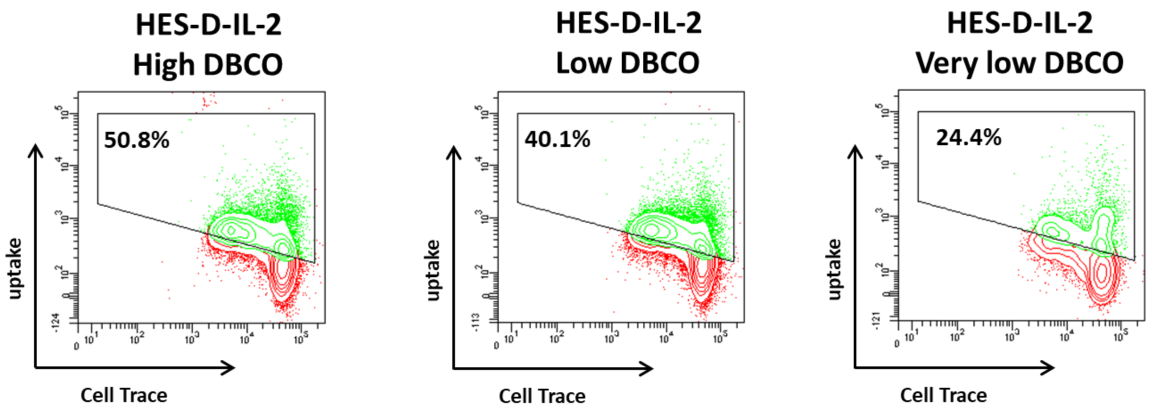

Figure S4: Human $CD4^{+}CD25^{+}$ T cells were incubated with 25 µg of high, low and very low DBCO HES-D-IL-2 (IL-2 ratio 1:1), respectively, and percentages of nanocapsule positive cells were measured by flow cytometry. The nanocapsule incorporated dye SR101 (for uptake/binding) (y-axis) was plotted against CellTrace Violet (x-axis). Dilution of CellTrace Violet shows IL-2 induced T cell proliferation. One representative experiment out of two is depicted.

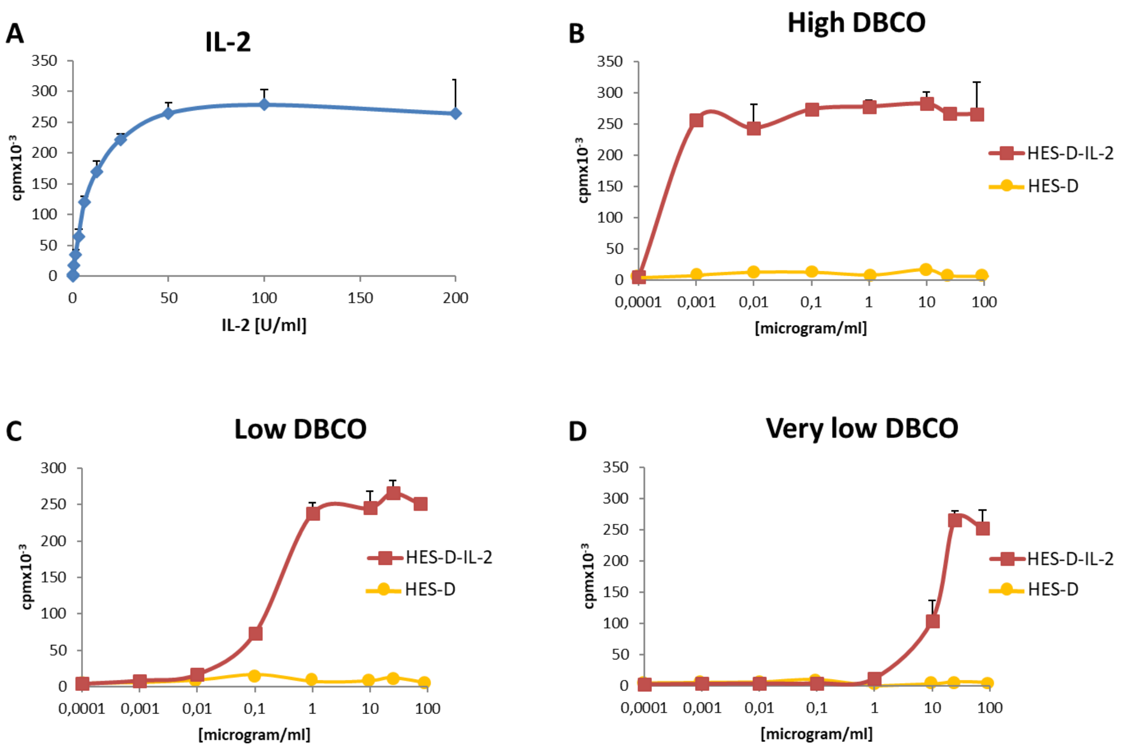

Figure S5: Proliferation of IL-2 dependent murine T cells (CTLL-2 cell line). (A) Control experiment showed the dose-dependent proliferation of CTLL-2 cells induced by human IL-2. HES-D and HES-D-IL-2 as high (B), low (C) and very low (D) DBCO variants (IL-2 ratio 1:1) were incubated with CTLL2 cells in concentrations as depicted. Proliferation was assessed by [^{3}H] thymidine incorporation, and counts per minute (cpm) were plotted against concentrations. One representative experiment out of 2 with mean ± SD from triplicates is demonstrated.

1. Frick, S.U.; Domogalla, M.P.; Baier, G.; Wurm, F.R.; Mailänder, V.; Landfester, K.; Steinbrink, K. IL-2 functionalized nanocapsules for T cell-based immunotherapy. *ACS Nano* **2016**, *10*, 9216–9226
